# Perillaldehyde combined with domiphen: synergistic bactericidal and anti-biofilm activity against *Staphylococcus aureus* and *Escherichia coli*

**DOI:** 10.3389/fcimb.2026.1769865

**Published:** 2026-03-10

**Authors:** Jiaju Qiao, Shengmin Wu, Cuiyan Fu, Quanlin Zhao, Yang Gong, Linjie Xu, Dandan Tang, Yuan Gao, Wanyi Luo

**Affiliations:** 1 Department of Biotechnology, School of Life Sciences, Xuzhou Medical University, Xuzhou, China; 2 Nanjing Institute of Environmental Sciences, Ministry of Ecology and Environment, Nanjing, Jiangsu, China

**Keywords:** biofilm, drug combination, foodborne pathogen, natural product, synergistic effect

## Abstract

**Introduction:**

Biofilms formed by pathogenic bacteria such as *Staphylococcus aureus* and *Escherichia coli* pose a significant threat to public health. Combination therapy has emerged as a promising strategy to combat bacterial infections and biofilm formation. In this study, the natural product perillaldehyde and the surfactant domiphen were evaluated for their ability to inhibit biofilm formation by these pathogenic strains.

**Methods:**

The antimicrobial activity of perillaldehyde and domiphen, alone and in combination, was assessed against *S. aureus* and *E. coli* strains. Synergism was determined by calculating the fractional inhibitory concentration index. Biofilm mass was evaluated using the crystal violet staining assay, and the viability of biofilm cells on stainless steel and polyethylene surfaces was examined via viable cell counting. Additionally, the therapeutic potential of the combination was further assessed using a *Galleria mellonella* larval infection model.

**Results:**

The combination of perillaldehyde and domiphen showed synergistic effects against both pathogenic strains, with a fractional inhibitory concentration index of less than 0.36. The combination of 1 μL/mL perillaldehyde and 1 μg/mL domiphen dispersed more than 53% of the biofilm mass in both *S. aureus* and *E. coli* strains. In addition, the combination reduced the total viable bacterial counts in biofilms on stainless steel and polyethylene surfaces by approximately 103 CFU/mL. The treatment also significantly improved the survival rate of *G. mellonella* larvae infected with the bacteria.

**Discussion:**

These results indicate that the novel combination of perillaldehyde and domiphen has the potential to decrease biofilm formation on various industrial material surfaces.

## 1 Introduction

Bacterial biofilms develop stronger adhesion, heightened pathogenicity, and increased resistance to antibiotics and phagocytosis (Xia et al., 2025). Biofilms contamination in public health engineering systems presents a serious risk to human health and industrial operations, leading to annual economic losses amounting to thousands of dollars (Xia et al., 2025).

*Staphylococcus aureus* forms biofilms on food-contact surfaces, including processing equipment and packaging materials, which contributes to its persistence (Bai et al., 2022). These biofilms can cause metal corrosion in pipelines and equipment, leading to increased production costs. More critically, the biofilm matrix provides a physical barrier that protects bacteria, enhancing their resistance to antimicrobials and increasing the risk of cross-contamination, which poses a severe threat to food safety (Qiao et al., 2024). In healthcare, biofilms on medical devices protect embedded microorganisms from host immunity and therapies, making them a common cause of persistent infections (Cacace et al., 2023). Notably, *S. aureus* and *S. epidermidis* are among the most prevalent biofilm-forming bacterial species.

*Escherichia coli*, a facultative anaerobic, Gram-negative bacterium, is a major global cause of foodborne illness (Kang et al., 2018). Biofilm formation enables *E. coli* to colonize diverse ecological niches, such as soil, water, vegetables, and agri-food surfaces, and to asymptomatically colonize certain hosts. These biofilm-associated bacteria can be transmitted through the food chain, ultimately leading to human infections (Ageorges et al., 2020).

Meanwhile, bacterial biofilms contribute significantly to antimicrobial resistance and are frequently associated with chronic and persistent infections. Bacterial biofilm matrices impede the penetration of conventional antibiotics through mechanisms such as steric hindrance, hydrophobic interactions, and π-π stacking (Hu et al., 2023). Therefore, it has become critically important to develop novel strategies to combat biofilms and drug-resistant bacteria (Hu et al., 2023). Antimicrobial resistance (AMR) constitutes a global public health threat, prompting growing interest in drug combinations. Multidrug combination therapy confers potential advantages, including broadening the spectrum of targeted pathogens, suppressing the emergence of antimicrobial resistance, and enhancing clinical efficacy (Lázár et al., 2022). The use of two or more antimicrobial agents, or the combination of therapeutics with adjuvants, represents a promising approach to overcome resistance and potentially eradicate bacterial biofilms (Hu et al., 2023).

Many essential oils are recognized as natural, safe food additives and preservatives, with the ability to effectively extend food shelf life. The FAO/WHO Joint Expert Committee on Food Additives (JECFA) has assessed perillaldehyde and deemed it safe (Benny et al., 2022). Perillaldehyde (PAE), a natural monoterpenoid compound, is extracted from the fruits of *Perilla frutescens* (Wang et al., 2022). It has been reported to exhibit diverse biological activities, including anti-inflammatory, antioxidant, and antibacterial properties. Mechanistically, PAE can disrupt microbial cell membranes by binding to bacterial or fungal proteins, thereby increasing cell membrane permeability. However, the effect of PAE on bacterial biofilm formation remains uninvestigated.

In this study, the natural product perillaldehyde and the surfactant domiphen were used in combination. This drug combination was employed for exerting antibacterial and anti-biofilm activities against *S. aureus* and *E. coli*. It was hypothesized that the surfactant could disperse the biofilm matrix, thereby enhancing the permeability of perillaldehyde within the biofilm and improving its bactericidal efficacy. The purpose of this study was to utilize this drug combination to reduce drug side effects, as well as to improve the removal efficiency of pathogenic bacterial biofilms on the surfaces of materials including stainless steel, polyethylene, and rubber.

## 2 Materials and methods

### 2.1 Materials

*S. aureus* ATCC 6538, and *E. coli* ATCC 25922 strains were purchased from the Chinese Center of Industrial Culture Collection. Perillaldehyde was obtained from Yuanye (Shanghai, China). Domiphen was purchased from ABM (Nanjing, China). The fluorescent stain SYTO and propidium iodide (PI) was obtained from KeyGen (Nanjing, China).

### 2.2 Antimicrobial susceptibility

The broth microdilution method was performed according to the guidelines of the Clinical and Laboratory Standards Institute (Qiao et al., 2020). *S. aureus* ATCC 6538 and *E. coli* ATCC 25922 were cultured at 37°C for 10 h to reach the logarithmic growth phase. Bacterial cell concentrations were adjusted to an optical density at 595 nm of 0.5 (approximately 10^8^ CFU/mL) and subsequently diluted to 10^5^ CFU/mL in Mueller–Hinton Broth (MHB). The maximum test concentrations were 64 µL/mL for perillaldehyde and 128 μg/mL for domiphen. Each agent was serially diluted two-fold in MHB to generate concentration gradients. The minimum inhibitory concentration (MIC) was determined after incubating the plates at 37 °C for 18 h. For minimum bactericidal concentration (MBC) assay, bacterial cultures were treated with each agent at concentrations equivalent to the MIC, 2×MIC, 4×MIC, 8×MIC, 16×MIC, and 32×MIC, then plated onto Luria-Bertani (LB) agar. The MBC was defined as the lowest concentration that resulted in no visible growth on the agar plates after incubation.

### 2.3 Antibacterial activity of the combination of perillaldehyde and domiphen

The synergistic antibacterial efficacy of perillaldehyde and domiphen was evaluated against *S. aureus* ATCC 6538 and *E. coli* ATCC 25922 using the checkerboard microdilution method. A series of concentrations of perillaldehyde (0.125, 0.25, 0.5, 1, 2, 4, 8 and 16 μL/mL) and domiphen (0.25, 0.5, 1, 2, 4, 8, 16 and 32 μg/mL) were co-administered in 96-well microtiter plates. Bacterial suspensions were adjusted to approximately 10^5^ CFU/mL. After incubation at 37 °C for 18 h, the MIC of the combination was determined. The MBC was assessed as described in Section 2.2.

The fractional inhibitory concentration (FIC) and fractional bactericidal concentration (FBC) indices were calculated based on the MIC and MBC values, respectively (Tamang et al., 2022). The FIC index (FICI) was defined by the formula:


FICI= (MICA'/MICA) + (MICB'/MICB)

where MIC_A_ and MIC_B_ represent the MICs of perillaldehyde and domiphen alone, respectively; MIC_A_’ and MIC_B_’ correspond to the MICs of each agent when used in combination.

Similarly, the FBC index (FBCI) was calculated as:


FBCI= (MBCA'/MBCA) + (MBCB'/MBCB)

where MBC_A_ and MBC_B_ denote the MBCs of perillaldehyde and domiphen alone, respectively; MBC_A_’ and MBC_B_’ refer to the MBCs of each agent in the combination.

Synergy was defined as a FICI or FBCI of ≤ 0.5. Additivity/indifference was defined as a FICI or FBCI of > 0.5 to ≤ 4. Antagonism was characterized by a FICI or FBCI of > 4 (Valcourt et al., 2016; Belguesmia et al., 2021). The synergistic effect was analyzed using GraphPad Prism 9.

### 2.4 Inhibition of biofilm formation

Biofilm formation was assessed using the crystal violet staining method (O’Toole et al., 1999). Briefly, *S. aureus* ATCC 6538 and *E. coli* ATCC 25922 cells were cultured in nutrient broth (NB) at 37°C for 10 h. Bacterial suspension (10^6^ CFU/mL) was added to 96-well microtiter plates at 200 µL per well. After incubation at 37°C for 1 h, the culture medium was aspirated, and non-adherent bacterial cells were removed by PBS (pH 7.2). Biofilms were then cultured in NB medium with different concentrations of perillaldehyde (0.13, 0.25, 0.5, 1, 2, 4, 8, 16 and 32 µL/mL) or domiphen (0.13, 0.25, 0.5, 1, 2 and 4 µg/mL) for 24 h at 37°C. The resulting biofilms were fixed with 200 μL of 0.5% crystal violet for 30 minutes. After staining, the wells were gently washed with sterile water to remove unbound dye. The crystal violet was then solubilized by adding 95% ethanol and incubating for 20 minutes. Finally, OD_595_ values were measured using a microplate reader to quantify the biofilm biomass.

The anti-biofilm activity of the drug combination was evaluated against *S. aureus* ATCC 6538, and *E. coli* ATCC 25922 strains using the checkerboard assay. Concentration gradients of perillaldehyde (0.5, 1, 2, 4, 8 and 16 µL/mL) and domiphen (0.5, 1, 2, 4, 8 and 16 µg/mL) were prepared. A mixture of 100 μL of drug solution and 100 μL of bacterial suspension (10^6^ CFU/mL) was added to each well of a 96-well plate. After 24 h of incubation at 37 °C (Sacks et al., 2018), the medium was discarded, and biofilm staining and quantification were performed as described in Section 2.4.

### 2.5 Clearance of mature biofilms assessed by viable bacterial counts

#### 2.5.1 Removal of biofilm on stainless steel surfaces

*S. aureus* ATCC 6538 and *E. coli* ATCC 25922 were cultivated to the mid-log phase at 37°C. The bacterial suspension (2 mL, 10^6^ CFU/mL) was added to a 6-well plate containing a stainless steel plate (Type 304, 18 ×18 × 2 mm^3^). The medium was discarded after incubating at 37 °C for 48 h and washed three times with sterile water. To remove the biofilm, 2.5 mL of a combination of perillaldehyde (1 μL/mL) and domiphen (1 μg/mL) was added for 4 h. Phosphate-buffered saline was added as the control group. After sonication, 1 mL of the microbial suspension was serially diluted, plated on LB agar, and incubated at 37 °C for 18 h. Finally, the total viable count of the biofilm cells after the treatment with antimicrobial reagents was calculated.

#### 2.5.2 Removal of biofilm on the surface of polyethylene material

*S. aureus* ATCC 6538 and *E. coli* ATCC 25922 strains were cultured at 37°C to mid-log phase. A 2 mL volume of microbial suspension (10^6^ CFU/mL) was added to a 6-well cell culture plate (35 mm diameter, 2 mm height). The treatment was similar to that described in section 2.5.1.

#### 2.5.3 Removal of biofilm on the surface of rubber

*S. aureus* ATCC 6538 and *E. coli* ATCC 25922 strains were grown to mid-log phase at 37 °C. The bacterial suspension (2 mL, 10^6^ CFU/mL) was added to a 6-well plate containing sterile rubber coupon (18 × 18 mm^2^). Biofilm removal was evaluated using the same methodology outlined in Section 2.5.1.

#### 2.5.4 Analysis of biofilm by fluorescence microscopy

To evaluate bacterial viability after drug combination removal, fluorescence microscopy was employed. The microbial biofilm was cultured for 48 h, washed to remove planktonic cells, and treated with a combination of perillaldehyde and domiphen for 4 h. The biofilms were stained using a mixture of 200 µL volume of 10 µM SYTO and 30 µM PI solution at room temperature in the dark for 30 min. The stained biofilm was then scanned using an Olympus IX73 fluorescence microscope (Kurz et al., 2021). ImageJ software was used to analyze fluorescence intensity.

### 2.6 Effectiveness of drug combination on the *Galleria mellonella*

*S. aureus* ATCC 6538 and *E. coli* ATCC 25922 strains were cultivated to the mid-log phase at 37°C. Forty *Galleria mellonella* larvae were selected and randomly assigned to four groups (n=10 per group): a physiological saline control group, a combination therapy group, a perillaldehyde-treated group, and a domiphen-treated group. Larvae were immobilized to expose their ventral side. A 10 μL aliquot of diluted bacterial suspension (10^7^ CFU/mL) was injected into the penultimate left limb using a microsyringe. One hour later, 10 μL of the assigned treatment was injected into the other right limb. For the uninfected control group, sterile water was substituted for the bacterial suspension. The larvae were maintained at 37 °C and monitored daily over a 7-day observation period. Individuals that were unresponsive to physical stimulation were recorded as dead (Chen et al., 2024).

### 2.7 Statistical analysis

Statistical analyses were performed using IBM SPSS Statistics 20 (New York, USA). All experiments were repeated at least three times. One-way analysis of variance was used for every figure, and the means were compared using Tukey’s multiple-range tests. Values are presented as means ± standard deviations. Statistical significance was set at *P*< 0.05, * indicates *P<* 0.05, and ** indicates *P<* 0.01, compared to the control group. The results were plotted using Origin 8.5 software.

## 3 Result

### 3.1 The combined effect of perillaldehyde and domiphen against bacteria

**Figure 1:**
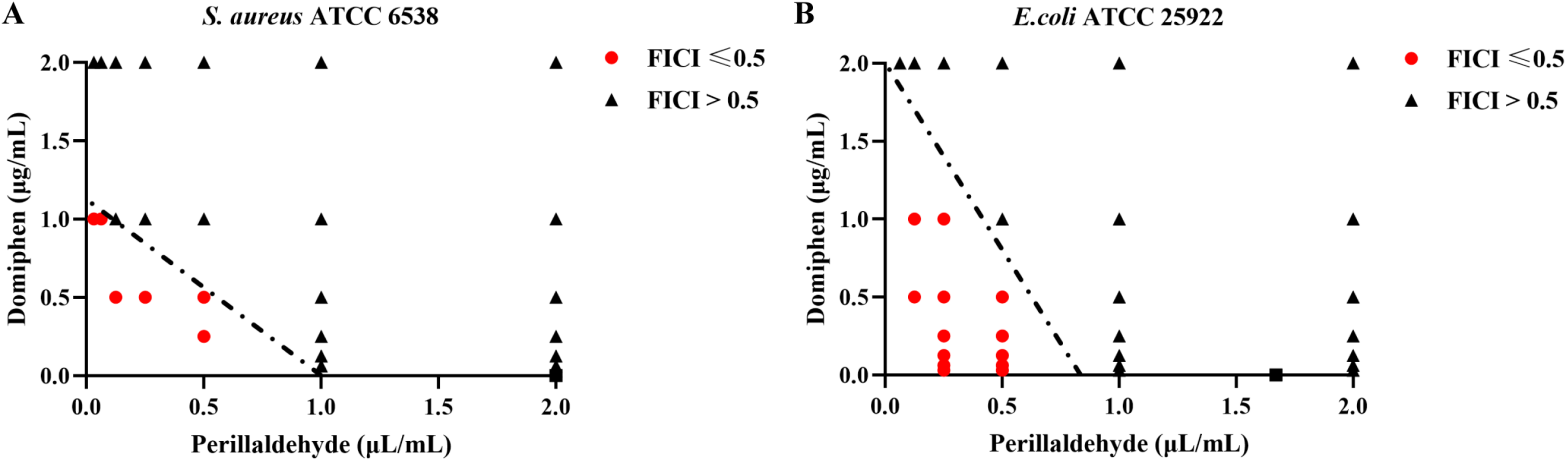
The combined effect of perillaldehyde and domiphen against bacteria Synergistic effects of perillaldehyde combined with domiphen against *S. aureus***(A)** and *E. coli***(B)**. The x-axis and y-axis show the concentrations of perillaldehyde and domiphen, respectively. The line of additivity, or synergy threshold, is indicated by a dashed line. Data points with FICI values of ≤0.5, indicating synergy, are marked in red and fall below this line.

The fractional inhibitory concentration (FIC) of perillaldehyde and domiphen against *S. aureus* and *E. coli* strains were determined using the checkerboard assay (**Figure 1**). Perillaldehyde exhibited MIC values of 2 μL/mL and 1.67 μL/mL against *S. aureus* ATCC 6538 and *E. coli* ATCC 25922, respectively (**Table 1**). Domiphen showed MIC values of 2.25 μg/mL and 4 μg/mL against the same strains.

**Table 1:** Synergistic bactericidal effect between perillaldehyde and domiphen.

Strains	MIC		MBC		MBC’		FBC
	Perillaldehyde (μL/mL)	Domiphen (μg/mL)	Perillaldehyde (μL/mL)	Domiphen (μg/mL)	Perillaldehyde (μL/mL)	Domiphen (μg/mL)	
*S. aureus* ATCC 6538	2	2.25	8	4	1	0.5	0.25
*E. coli* ATCC 25922	1.67	4	8	8	0.5	2	0.315

MIC, minimum inhibitory concentration; MBC, minimum bactericidal concentration; MBC’, the MBC of perillaldehyde in combination with domiphen; FBC, The fractional bactericidal concentration. FBC< 0.5 indicates a synergistic effect.

The combination of perillaldehyde and domiphen exhibited enhanced antimicrobial activity against both strains compared to each agent alone, resulting in lower effective doses (**Figure 1**). This synergy against *S. aureus* (**Figure 1A**) was evident at specific concentration ratios: 0.25 μL/mL perillaldehyde to 0.5 μg/mL domiphen (FICI = 0.347), 0.5 μL/mL to 0.25 μg/mL (FICI = 0.361), 0.5 μL/mL to 0.5 μg/mL (FICI = 0.472), 0.063 μL/mL to 1 μg/mL (FICI = 0.476), and 0.032 μL/mL to 1 μg/mL (FICI = 0.46). Similarly, for *E. coli* (**Figure 1B**), synergistic antibacterial effects were observed across a series of combinations. Representative examples include: 0.125 μL/mL perillaldehyde + 1 μg/mL domiphen (FICI = 0.325), 0.25 μL/mL + 0.5 μg/mL (FICI = 0.275), and 0.5 μL/mL + 0.25 μg/mL (FICI = 0.362). In particular, when perillaldehyde was used at 0.5 or 0.25 μL/mL in combination with domiphen (ranging from 0.0625 to 0.5 μg/mL), the resulting FICI values consistently indicated synergy.

To further evaluate the efficacy of the combination of perillaldehyde and domiphen, the minimum bactericidal concentration (MBC) was determined. The MBC values for *S. aureus* and *E. coli* were 1 μL/mL perillaldehyde + 0.5 μg/mL domiphen and 0.5 μL/mL perillaldehyde + 2 μg/mL domiphen, respectively (**Table 1**). The corresponding fractional bactericidal concentration (FBC) indices were 0.25 and 0.315, indicating a synergistic bactericidal effect between the two agents.

### 3.2 Efficacy of the perillaldehyde and domiphen combination against bacterial biofilms

**Figure 2:**
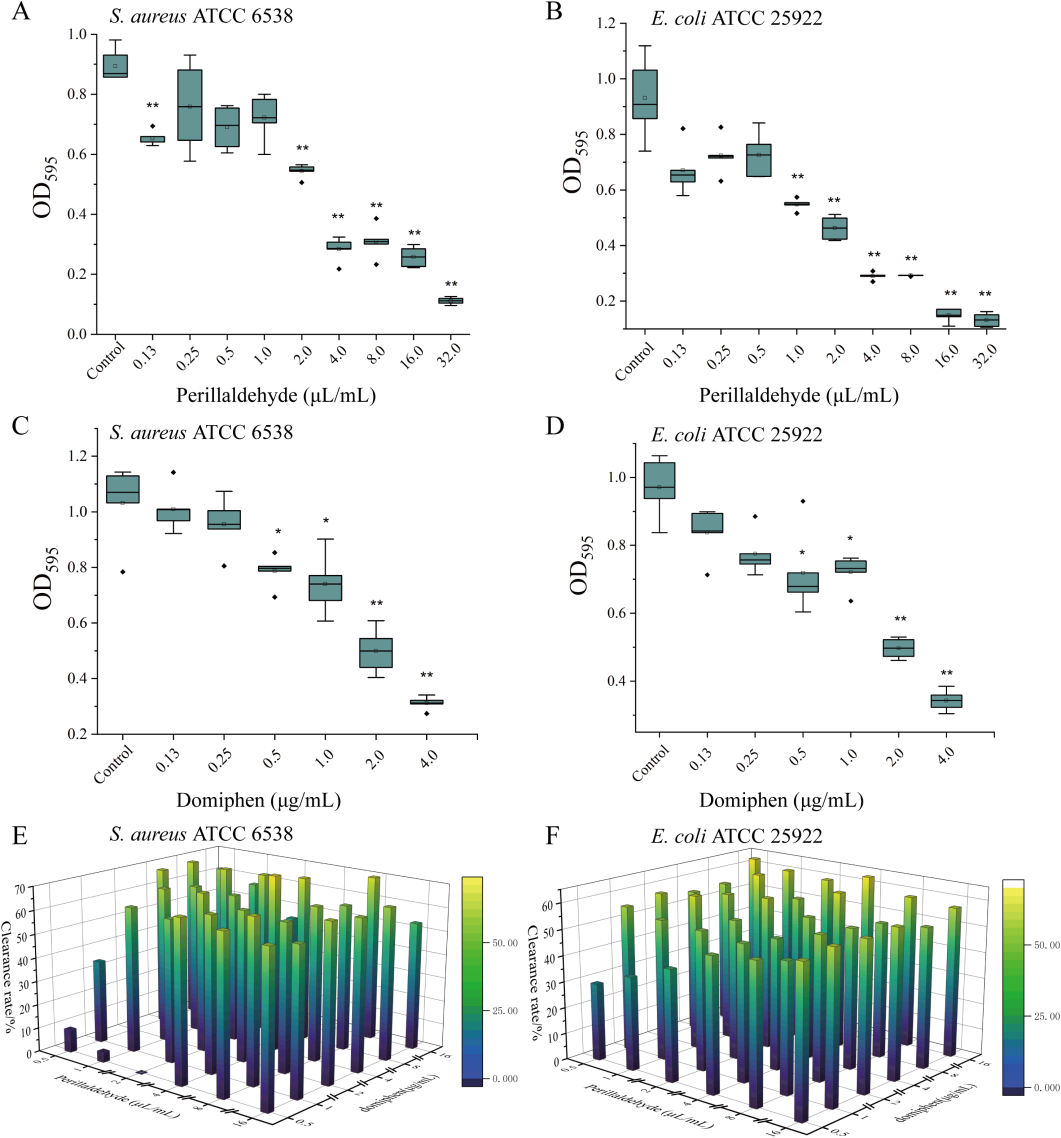
Anti-biofilm effects in various experimental groups. The inhibitory effect of perillaldehyde against biofilms of *S. aureus* ATCC 6538 **(A)** and *E. coli* ATCC 25922 **(B)**. The inhibitory effect of domiphen against biofilms of *S. aureus* ATCC 6538 **(C)** and *E. coli* ATCC 25922 **(D)**. Combination of perillaldehyde and domiphen against biofilms of *S. aureus* ATCC 6538 **(E)** and *E. coli* ATCC 25922 **(F)**. Data are presented as means ± standard deviation. Compared to the control group, *indicates *P*< 0.05, and **indicates *P*< 0.01.

Assessed by crystal violet staining for biofilm biomass, the efficacy of perillaldehyde in inhibiting the biofilm formation of *S. aureus* ATCC 6538 and *E. coli* ATCC 25922 was enhanced with increasing concentration. Compared with the biofilm formation of *S. aureus* and *E. coli* cells in the control group, those in the groups treated with perillaldehyde at a concentration exceeding 2 μL/mL were significantly reduced (**Figures 2A, B**, *P*< 0.01). Furthermore, perillaldehyde at a concentration of 4 μL/mL effectively decreased their biofilm biomass by more than 50% (*P<* 0.01). Moreover, 4 μg/mL domiphen effectively reduced the biofilm biomass of these two strains by more than 50% (**Figures 2C, D**, *P<* 0.01).

The anti-biofilm efficacy of the perillaldehyde-domiphen combination was further investigated. Specifically, 1 μL/mL perillaldehyde combined with 1 μg/mL domiphen reduced the biofilm biomass of *S. aureus* and *E. coli* cells by 65% and 53%, respectively (*P<* 0.01, **Figures 2E, F**). Thus, in combination, the effective dosage of each agent is reduced to one-fourth of that required in monotherapy, significantly lowering the dosage of both agents.

### 3.3 Biofilm removal activity

To evaluate the biofilm removal efficacy of the perillaldehyde and domiphen combination under practical conditions, the total viable count was assessed on three common surfaces: stainless steel, polyethylene, and rubber.

#### 3.3.1 Using drug combination for removing bacterial biofilms on stainless steel surfaces

**Figure 3:**
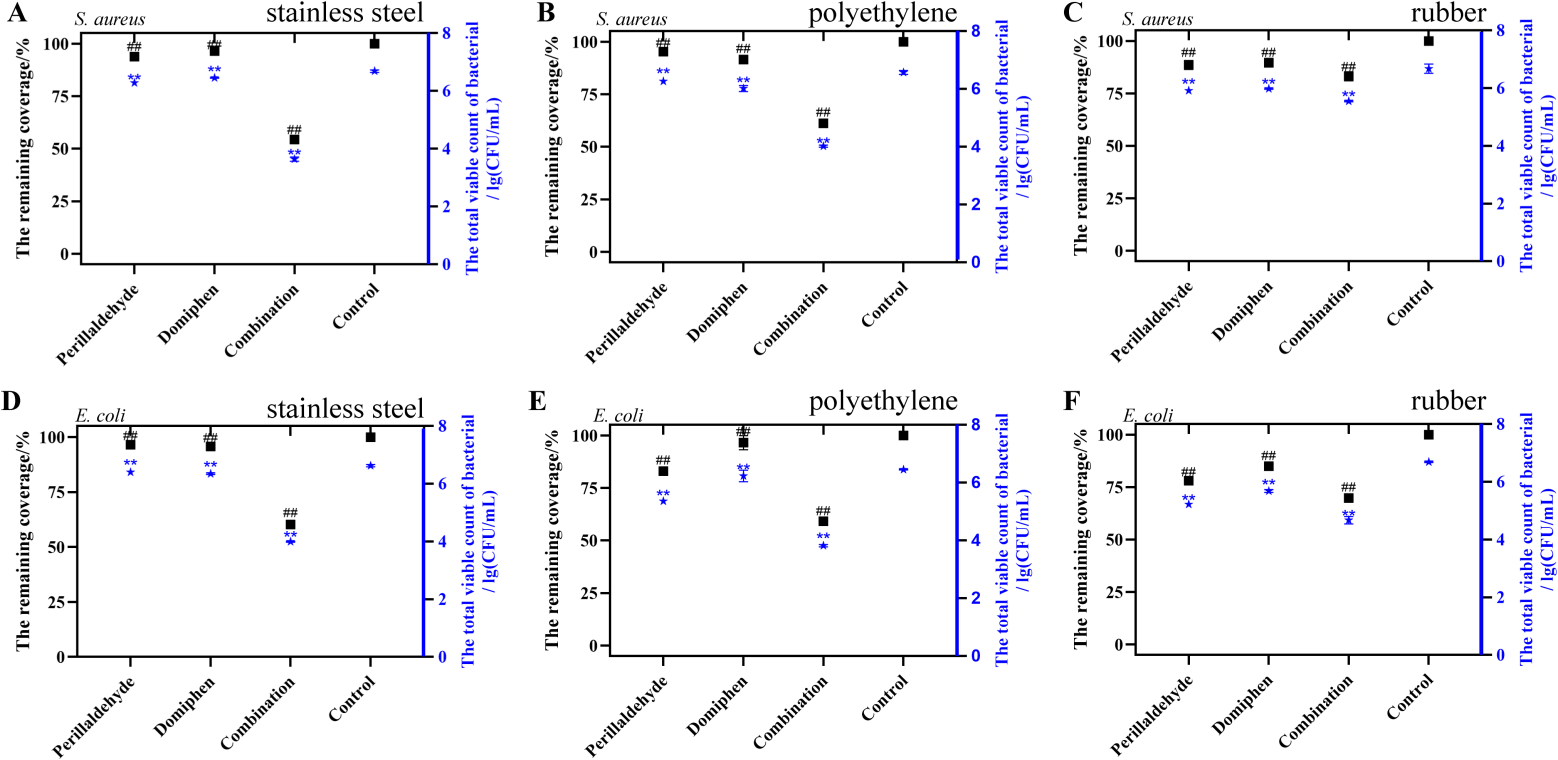
Biofilm removal effects in various experimental groups. Removal of *S. aureus* ATCC 6538 **(A)** and *E. coli* ATCC 25922 **(D)** biofilms from the stainless steel surface using different reagents. Removal of *S. aureus***(B)** and *E. coli***(E)** biofilms from polyethylene materials surfaces. Removal of *S. aureus***(C)** and *E. coli***(F)** biofilms from rubber surfaces. Combination: a combination of perillaldehyde (1 μL/mL) and domiphen (1 μg/mL). Data are presented as means ± standard deviation. Compared to the control group, ** indicates *P*< 0.01. Significant differences compared to the control group are indicated as follows: “##” P < 0.01 for the remaining coverage; “**” P < 0.01 for the total viable bacterial count.

The initial viable count of *S. aureus* ATCC 6538 (**Figure 3A**) and *E. coli* ATCC 25922 (**Figure 3D**) biofilms on stainless steel surfaces were 4.9×10^6^ CFU/cm³ and 4.3×10^6^ CFU/cm³, respectively. Treatment with a combination of perillaldehyde (1 μL/mL) and domiphen (1 μg/mL) significantly reduced these counts to 4.45×10³ CFU/cm³ for *S. aureus* and 9.9×10³ CFU/cm³ for *E. coli*, corresponding to eradication efficiencies of 46.28% and 40.38%, respectively. In comparison, either agent used alone resulted in less than a 10-fold reduction and an eradication efficiency below 6.09%. Notably, the combination of perillaldehyde and domiphen reduced the viable counts of *E. coli* and *S. aureus* biofilm cells by approximately 10^3^ CFU/mL (*P*< 0.01).

#### 3.3.2 Using drug combination for removing bacterial biofilms on polyethylene material surfaces

Compared with the control group, the combination of 1 μL/mL perillaldehyde and 1 μg/mL domiphen reduced the viable counts in *S. aureus* ATCC 6538 (**Figure 3B**) and *E. coli* ATCC 25922 (**Figure 3E**) biofilms on the surface of polyethylene materials by approximately 10^3^ CFU/mL, corresponding to an eradication efficiency of approximately 40.76% (*P<* 0.01). Furthermore, this combined treatment achieved a reduction greater than 10² CFU/mL relative to either agent administered alone, exhibiting a highly significant difference (*P*< 0.01).

#### 3.3.3 Using drug combination for removing bacterial biofilms on rubber surfaces

On rubber surfaces, the combination of perillaldehyde (1 μL/mL) and domiphen (1 μg/mL) significantly reduced viable *S. aureus* biofilm counts by approximately 10^2^ CFU/mL compared to the control (**Figure 3C**, P< 0.01), corresponding to a 30.13% eradication efficiency. Against *E. coli* biofilms, the same combination achieved a significant 10-fold decrease, representing a 14.42% eradication efficiency (**Figure 3F**, *P*< 0.01). In contrast, perillaldehyde monotherapy demonstrated eradication rates of less than 21.89% against both *S. aureus* and *E. coli* biofilms, while the eradication rates for domiphen monotherapy were all below 9.25%.

### 3.4 Observation of biofilm removal by drug combination

**Figure 4:**
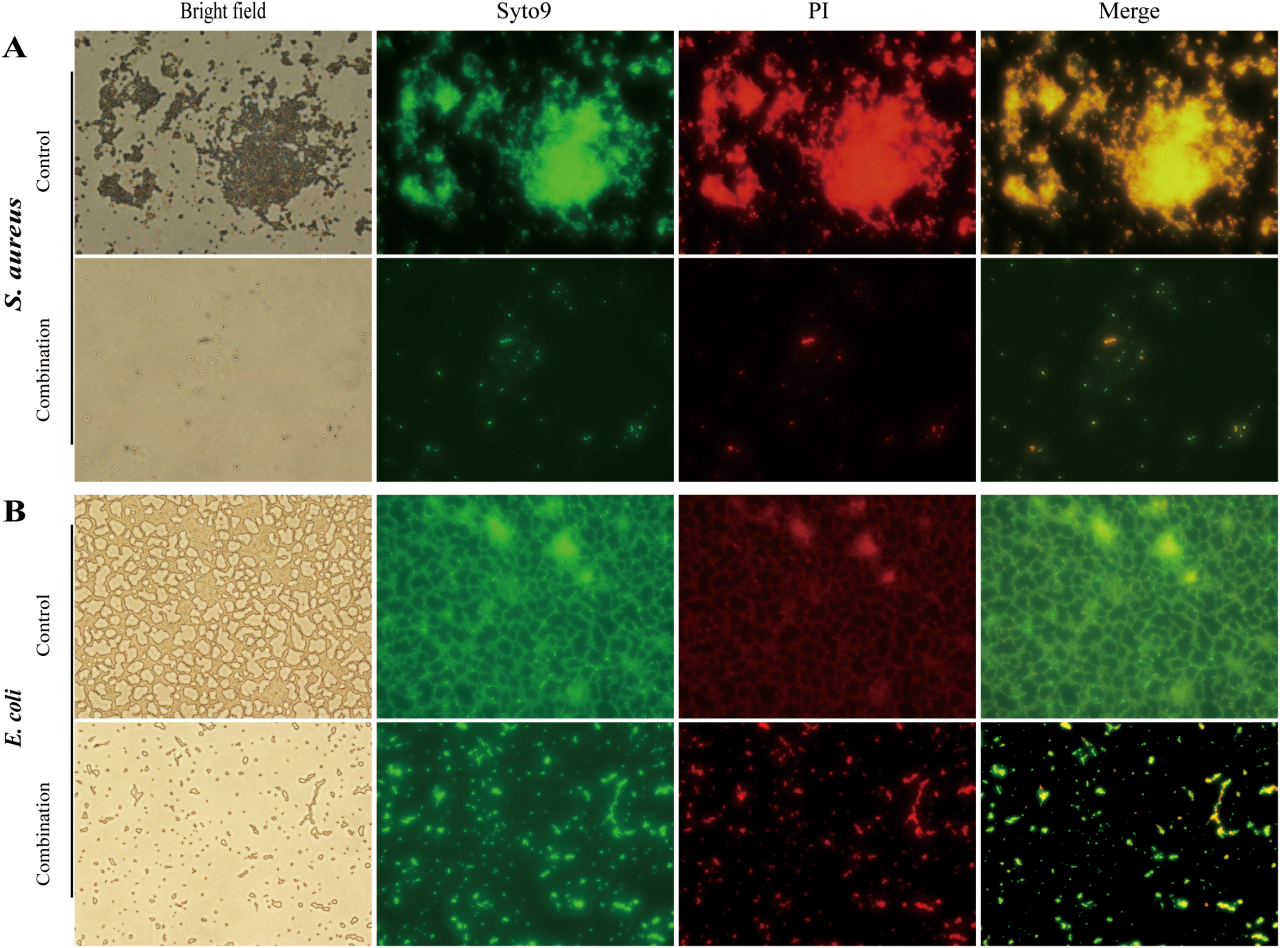
Characterization of bacterial biofilm removal by drug combination. FM images of *S. aureus* ATCC 6538 **(A)** and *E. coli* ATCC 25922 **(B)** biofilms with the combination of perillaldehyde (1 μL/mL) and domiphen (1 μg/mL). Images correspond to bright field, Syto9 (living cells with green fluorescent), PI (dead cells with red fluorescence), and merged image of bacterial biofilm.

The biofilms of *S. aureus* ATCC 6538 (**Figure 4A**) and *E. coli* ATCC 25922 (**Figure 4B**) in the untreated group were dense and thick, and the bacterial cells within these biofilms exhibited distinct green fluorescence (characteristic of viable bacteria). After treatment with the combination of perillaldehyde (1 μL/mL) and domiphen bromide (1 μg/mL), the biofilm mass of both *S. aureus* and *E. coli* strains was effectively dispersed. Additionally, the biofilm cells of these two strains were killed, resulting in noticeably thinner biofilms. Under this combined treatment, dead bacterial cells—marked by red fluorescence (typical of dead bacteria)—were easily observed. Collectively, the drug combination not only effectively dispersed the biofilms of *S. aureus* and *E. coli* but also significantly reduced the viability of their biofilm-associated cells.

### 3.5 Effectiveness of drug combination on the *Galleria mellonella*

**Figure 5:**
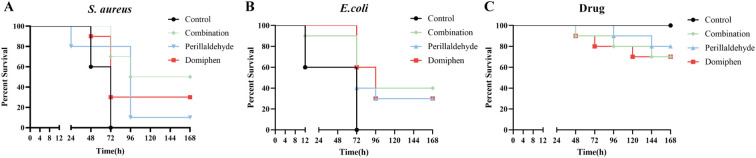
Effectiveness of drug combination on the *Galleria mellonella G. mellonella* larvae were infected with *S. aureus* ATCC 6538 **(A)** and *E. coli* ATCC 25922 **(B)**. **(C)** Effect of drug treatment in the absence of bacterial infection. The infected larvae were then subjected to therapeutic treatment. Survival rates were monitored for over 168 hours post-treatment. Combination: a combination of perillaldehyde (1 μL/mL) and domiphen (1 μg/mL).

To further investigate the effect of drug treatments on the survival rate of *Galleria mellonella* infected with bacteria, the *G. mellonella* infection model was employed to monitor the survival of *G. mellonella* over a 168-hour period. For infections caused by *S. aureus* ATCC 25922 (**Figure 5A**), the control group showed a 0% survival rate of *G. mellonella* after 72 hours. From 96 to 168 hours, the perillaldehyde-treated group maintained a 10% survival rate, and the domiphen-treated group showed a 30% survival rate. However, the combined treatment group sustained a high survival rate of 50%.

For infections caused by *E. coli* ATCC 25922 (**Figure 5B**), the survival rate of *G. mellonella* in the control group dropped to 0% after 72 hours. From 96 to 168 hours, the perillaldehyde-treated group and domiphen-treated group maintained a 30% survival rate. Notably, the combined treatment group retained a high survival rate of 40% over the same time period.

For the uninfected control group (**Figure 5C**), domiphen monotherapy reduced the survival rate of *G. mellonella* to 70% at 120 h; perillaldehyde monotherapy lowered this rate to 80% at 144 h; combined treatment with domiphen and perillaldehyde reduced the rate to 70% at 144 h.

## 4 Discussion

*Staphylococcus aureus* has emerged as the second most prevalent bacterial pathogen, second only to *Escherichia coli*. Staphylococcal enterotoxinsare responsible for approximately 33% of bacterial foodborne illness outbreaks in the United States, while this proportion exceeds 45% in Canada. Similarly, foodborne outbreaks caused by these toxins occur annually in China (Fang et al., 2024). Meanwhile, biofilms formed by *S. aureus* and *E. coli* induce contamination in the food industry (Pang et al., 2022), on medical devices (Liao et al., 2025), and in natural environment (Ormsby et al., 2024)—posing a substantial threat that has garnered widespread attention. Studies have demonstrated that pathogenic bacterial biofilms exhibit 10- to 1000-fold higher antibiotic tolerance (Zhang et al., 2025). As a result, a variety of alternatives to traditional chemical disinfectants are continuously being explored and developed (Shao et al., 2020). Moreover, combination drug therapy emerges as a promising strategy, as it offers the potential to enhance therapeutic efficacy, reduce the required drug dosage, minimize side effects, and overcome drug resistance (Babaei et al., 2023; Zhu et al., 2023).

Studies have shown that perillaldehyde exhibits varying degrees of inhibitory activity against pathogenic microorganisms, such as *Acinetobacter baumannii*, *Pseudomonas aeruginosa* (Wang et al., 2025), *Enterococcus faecium*, *Klebsiella pneumoniae*, *Candida albicans*, *Cryptococcus neoformans*, and *Fusarium solani* (Pan et al., 2023). Previous studies have not fully elucidated the activity of perillaldehyde against Gram-positive *S. aureus*. This work confirms its potent antibacterial effect (MIC = 2 μL/mL) but also reveals that a high concentration (8 μL/mL) is required for a bactericidal outcome. To address this limitation, we investigated its combination with domiphen. For the first time, synergistic antibacterial and bactericidal effects were demonstrated using specific ratios against both *S. aureus* and *E. coli*. Perillaldehyde-based combination therapies for biofilm inhibition are rarely reported. One study documented synergistic antimicrobial activity of lemongrass and perillaldehyde but did not investigate biofilm-related effects (Ishijima et al., 2021). Natural aldehydes as antibiotic adjuvants for biofilm-targeted therapy are also understudied (Wang et al., 2026), and our work fills this gap by targeting both planktonic bacteria and *S. aureus*/*E. coli* biofilms.

It has been demonstrated that perillaldehyde kills *Acinetobacter baumannii* by disrupting the bacterial cell membrane, a process that alters membrane permeability and leads to the leakage of intracellular proteins and polysaccharides (Chu et al., 2024). Specifically, perillaldehyde compromises the structure and function of the bacterial cell membrane, particularly disrupting the proton gradient. This disruption results in the failure of extracellular acidification, loss of membrane integrity, and ultimately bacterial cell death (Chen et al., 2020). Domiphen achieves rapid, broad-spectrum bactericidal activity primarily by physical disrupting the bacterial cell membrane, with its bactericidal effect further enhanced by the inhibition of energy metabolism and induction of oxidative stress (Chen et al., 2024). Additionally, against specific pathogens—such as *Aspergillus fumigatus*, a common pathogenic fungus—it exhibits targeted intracellular antimetabolic activity (Hu et al., 2021). In contrast to previously reported domiphen-based combinations, which involve miconazole (Tits et al., 2020), colistin (Chen et al., 2024) and AgNPs (Hu et al., 2021), perillaldehyde-domiphen pairing is the first to combine domiphen with a natural plant-derived aldehyde. The synergistic characteristics of the multiple antibacterial mechanisms between perillaldehyde and domiphen endow them with more pronounced advantages when applied in drug combination. Thus, the combination of these two agents holds promise as a potential strategy for overcoming drug resistance.

Notably, perillaldehyde and domiphen act synergistically to reduce the biomass of *S. aureus* and *E. coli* biofilms. It is important to acknowledge that the crystal violet staining method used to quantify biofilm biomass cannot distinguish between a reduction due to actual biofilm matrix disruption and that resulting from bacterial killing (Haney et al., 2021). To address this limitation and further verify the combined effect on viable cells within the biofilm, viable bacterial count assays were performed. The results confirm that the synergistic interaction significantly reduces the effective dosage of each agent required compared to their individual applications. Regarding the mechanisms, perillaldehyde alone has been applied to prevention of biofilm formation by *Shewanella putrefaciens* (Zhu et al., 2025), *C. albicans* (Chu et al., 2024), and *P. aeruginosa* (Benny et al., 2022). It interferes with biofilm formation through inhibiting the production of extracellular polymeric substances (EPS), markedly disrupting swarming motility (Benny et al., 2022), and suppressing bacterial quorum sensing (Chu et al., 2024). Domiphen is classified as a member of cationic surfactants, which are referred to as quaternary ammonium compounds, and is commonly employed as a surface disinfectant (Li et al., 2024). Surfactants facilitate the dissolution of EPS (He et al., 2022). Existing studies have demonstrated that the addition of domiphen to miconazole enables the eradication of *C. albicans* biofilms (Tits et al., 2020); The colistin/domiphen combination provides a promising strategy for addressing clinical challenges linked to *P. aeruginosa* biofilms-associated infections (Chen et al., 2024). In the present study, fluorescence microscopy observations confirmed that the combination of domiphen and perillaldehyde effectively dispersed the bacterial biofilm matrix. Building on the established biofilm-modulating properties of each agent, prior evidence of domiphen-mediated EPS disruption, and the phenotypic biofilm dispersion observed here, it is hypothesized that domiphen disrupts the biofilm matrix. This may improve the diffusion and penetration of perillaldehyde into deep biofilm layers, thereby significantly reducing biofilm biomass. Notably, this mechanistic explanation remains speculative at present, as direct quantitative analysis of EPS, bacterial membrane integrity assays, and biofilm permeability measurements were not performed in this study. Further investigations focusing on these key aspects are warranted to fully elucidate the precise molecular and structural mechanisms underlying the observed synergistic activity.

Meanwhile, this drug combination was applied to eradicate bacterial biofilms on the surfaces of stainless steel (Zhou et al., 2022), polyethylene material (Bao et al., 2022), and rubber material (Bao et al., 2022). These materials are commonly used in industry settings, medical devices, and environment surfaces. Bacteria tend to adhere to their surfaces and form biofilms, and the threat posed by this phenomenon to public health and safety has attracted widespread attention (Krsmanovic et al., 2021) (Anamul Hasan Chowdhury et al., 2024). The results demonstrated that the drug combination significantly reduced the number of viable bacteria in biofilms on stainless steel and polyethylene surfaces by 10^3^ CFU/mL. In addition to dispersing the biofilm matrix, the combination exerted a synergistic bactericidal effect, thereby mitigating recurrent biofilm contamination. However, bacterial biofilms on rubber surfaces were more resistant to eradication—a finding that may be attributed to the porous, deformable surface structure of rubber. These results highlight the need for proper storage of rubber items during use and the minimization of excess rubber waste, so as to reduce cross-contamination arising from biofilm dissemination (Anamul Hasan Chowdhury et al., 2024).

Given the promising *in vitro* synergistic antibacterial and biofilm-eradicating activities of the perillaldehyde-domiphen combination, we sought to validate its efficacy in an *in vivo Galleria mellonella* infection model (Chen et al., 2024; Woodford et al., 2025). The results demonstrated that the survival rate of uninfected larvae remained at 70% or higher following treatment with the tested drugs either alone or in combination, and the combined treatment significantly enhanced the survival rate of larvae infected with pathogenic bacteria. Notably, perillaldehyde is approved for use as a food additive (Wang et al., 2022). Domiphen has been extensively applied in clinical practice and is characterized by low cost and high availability (Chen et al., 2024). However, assessing clinical relevance also requires determining the degree to which these effects hold true for other bacterial species and within mammalian *in vivo* models.

A notable limitation of this study is the use of only a single reference strain each for *S. aureus* and *E. coli*, which restricts the generalizability of our findings to clinical isolates, including multidrug-resistant strains. Future studies will incorporate a diverse panel of clinical isolates to validate and extend these results.

## 5 Conclusion

In conclusion, the combination of perillaldehyde and domiphen exhibits synergistic antibacterial and anti-biofilm activity against *Staphylococcus aureus* and *Escherichia coli*. A specific combination of 1 μL/mL perillaldehyde and 1 μg/mL domiphen demonstrated potent efficacy in eradicating pre-formed biofilms on stainless steel and polyethylene surfaces. These findings underscore the potential of this combination as a promising strategy for controlling surface contamination mediated by bacterial biofilms across various industrial materials.

## Data Availability

The raw data supporting the conclusions of this article will be made available by the authors, without undue reservation.
